# Supplementary material for: The Evolution and Characterization of the RNA Interference Pathways in Lophotrochozoa
Source: Genome Biol Evol. 2024 May 7;16(5):evae098. doi: 10.1093/gbe/evae098 (PMC11114477; doi:10.1093/gbe/evae098)
Supplement: evae098_Supplementary_Data [file evae098_supplementary_data.zip › Supplementary_materials.zip!/selected_miRNAs/NoGe_selected_miRNAs/mirdeep_pdf_files/NMRB01002664.1_Notospermus_geniculatus_strain_Ushimado_scaffold2664,_whole_genome_shotgun_sequence_39911.pdf]

Provisional ID	: NMRB01002664.1_Notospermus_geniculatus_strain_Ushimado_scaffold2664,_whole_genome_shotgun_sequence_39911
Score total	: 2359.8
Score for star read(s)	: 3.9
Score for read counts	: 2350.9
Score for mfe	: 2
Score for randfold	: 0
Score for cons. seed	: 3
Total read count	: 4623
Mature read count	: 4485
Loop read count	: 0
Star read count	: 138

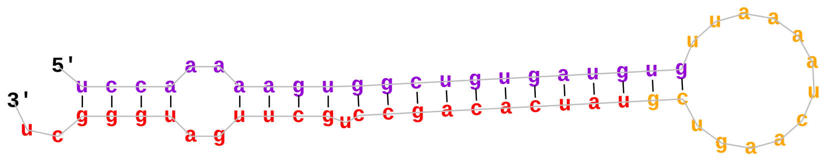

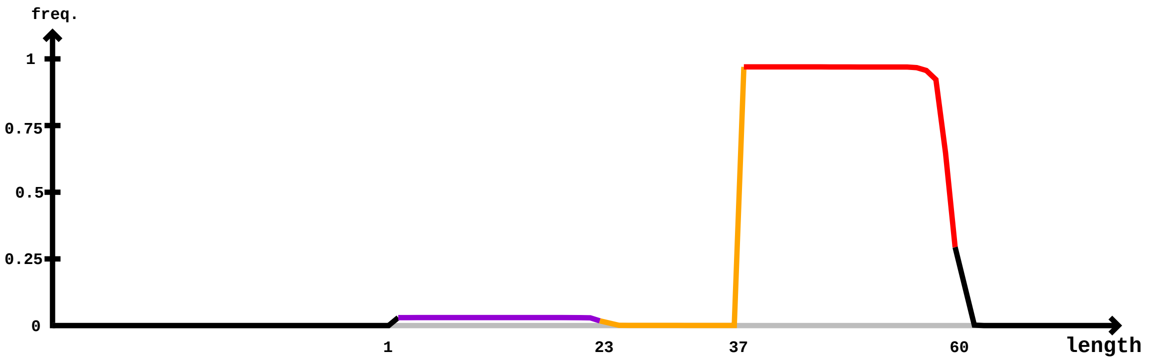

Star **Mature**

```
5'- ugguguacuagccgugcauuguauugcuucguauuccaaaaaguggcugugauguguuaaaaucaagucguaucacagccugcuugaugggcuauguggcaauacucuu  -3'  obs
    ugguguacuagccgugcauuguauugcuucguauuccaaaaaguggcugugauguguuaaaaucaagucguaucacagccugcuugaugggcuauguggcaauacucuu       exp
    .(((((((.....)))))))((((((((.((((.((((..((((((((((((((((............)))))))))))).))))..)))).)))).))))))))....  reads  mm  sample
    ..................................uccaaaaaguggcugugUugu......................................................  1      1   Fm1
    ......................................................................uaucacagccugcuugaug....................  3      0   Fm1
    ......................................................................uaucacagccugcuugaugg...................  6      0   Fm1
    ......................................................................uaucacagccugcuugauggg..................  95     0   Fm1
    ......................................................................uaucacagccugcuugGugggc.................  1      1   Fm1
    ......................................................................uauGacagccugcuugaugggc.................  1      1   Fm1
    ......................................................................uaucacagccuCcuugaugggc.................  1      1   Fm1
    ......................................................................uaucacagccugcuugCugggc.................  1      1   Fm1
    ......................................................................uaucacagccugcuugaugggA.................  12     1   Fm1
    ......................................................................uaucacagccugcuugaugAgc.................  7      1   Fm1
    ......................................................................uaucacagccugcuugaugggU.................  3      1   Fm1
    ......................................................................uaucacagccugcuugaGgggc.................  1      1   Fm1
    ......................................................................uaucacagccugcuugaAgggc.................  1      1   Fm1
    ......................................................................uaucacagccugcuugaugggc.................  171    0   Fm1
    ......................................................................Naucacagccugcuugaugggc.................  1      1   Fm1
    ......................................................................uaucacagccugcuugaCgggcu................  1      1   Fm1
    ......................................................................uaucacagccugcuugaugggAu................  1      1   Fm1
    ......................................................................uaucacagccugcuugaugggcG................  7      1   Fm1
    ......................................................................uaucacagccugcuugaugggcA................  1      1   Fm1
    ......................................................................uaucacagccugcuugaugggcu................  90     0   Fm1
    ......................................................................uaucacagccugcuugaugggcuU...............  6      1   Fm1
    ......................................................................uaucacagccugcuugaugggcuUu..............  4      1   Fm1

    ..................................uccaaaaaguggcugugaugu......................................................  11     0   Fm2
    ..................................uccaaaaaguAgcugugaugu......................................................  1      1   Fm2
    ..................................uccaaaaaguggcugugaugug.....................................................  29     0   Fm2
    ......................................................................uaucacagccugcuuUau.....................  5      1   Fm2
    ......................................................................uaucacagccugcuugaugg...................  20     0   Fm2
    ......................................................................uaucacagccugcuuAaugg...................  1      1   Fm2
    ......................................................................uaucacagccugcuugauggg..................  96     0   Fm2
    ......................................................................uaucacagccugcuugUuggg..................  1      1   Fm2
    ......................................................................uaucacagccugcuugauggU..................  1      1   Fm2
    ......................................................................uaucaAagccugcuugauggg..................  1      1   Fm2
```

Star Mature

```
ugguguacuagccgugcauuguauugcuucguauuccaaaaaguggcugugaugugguuaaaaucaagucguaucacagccugcuugaugggcuauguggcaauacucuu
......................................................................uaucacagccugcuugaAggg..................   1    1    Fm2
......................................................................uaucacagccugcuugaCggg..................   1    1    Fm2
......................................................................uaucacagccugcuugaugggc.................   20   0    Fm2
......................................................................uaucacagccugcuugaugggA.................   18   1    Fm2
......................................................................uaucacagccugcuugaugAgc.................   6    1    Fm2
......................................................................uaucacagccugcuugaugCgcu................   1    1    Fm2

..................................uccaaaaaguggcugugau........................................................   1    0    Mm2
..................................uccaaaaaguggcugugaugu......................................................   2    0    Mm2
..................................uccaaaaaguggcugugauguU.....................................................   1    1    Mm2
..................................uccaaaaaguggcugugaugug.....................................................   11   0    Mm2
..................................uccaaaaaguggcugugaugugu....................................................   3    0    Mm2
..................................uccaaaaaguggcugugauguguuaaaauc.............................................   1    0    Mm2
..........................................................aaaaucaagucguaucacagccug...........................   1    0    Mm2
......................................................................uaucacagccugcuugau.....................   1    0    Mm2
......................................................................uaucacagccugcuugauU....................   1    1    Mm2
......................................................................uaucacagccugcuugaGg....................   1    1    Mm2
......................................................................uaucacagccugcuugaug....................   7    0    Mm2
......................................................................uaucacagccugcuugaugg...................   11   0    Mm2
......................................................................uaucacagccugcuugaugU...................   2    1    Mm2
......................................................................uaucacagccugcuugUuggg..................   1    1    Mm2
......................................................................uaucacagccugcuugaugAg..................   1    1    Mm2
......................................................................uaucacagccugcuugaugUg..................   1    1    Mm2
......................................................................uaucacagccugcuugaCggg..................   1    1    Mm2
......................................................................uaucacagccugcuugauggg..................   203  0    Mm2
......................................................................uaucacagccugcuugaGggg..................   2    1    Mm2
......................................................................uaucacagccugcuugauggC..................   2    1    Mm2
......................................................................uaucacagccugcuugaAggg..................   1    1    Mm2
......................................................................uaucacagccugUuugauggg..................   1    1    Mm2
......................................................................uaucacagccAgcuugauggg..................   1    1    Mm2
......................................................................uaucacagccugcuugauCggc.................   1    1    Mm2
......................................................................uaucacagccugcuugaAgggc.................   1    1    Mm2
......................................................................uaucacagccugcuugUugggc.................   1    1    Mm2
......................................................................uaucacagccugcuugaugggU.................   4    1    Mm2
......................................................................uaucacagccugcuugaugggG.................   2    1    Mm2
......................................................................uaucacagccugcuCgaugggc.................   1    1    Mm2
......................................................................uaucacagccugcuugaugggA.................   21   1    Mm2
......................................................................uGucacagccugcuugaugggc.................   1    1    Mm2
......................................................................uaucacagccugcuugaugAgc.................   6    1    Mm2
......................................................................uaucacagccugcuugaugggc.................   353  0    Mm2
......................................................................uaucacagccugcuugCugggc.................   1    1    Mm2
......................................................................uaucacagccugcuugaugggAu................   4    1    Mm2
......................................................................uaucacagccugcuugaGgggcu................   1    1    Mm2
......................................................................uaGcacagccugcuugaugggcu................   1    1    Mm2
......................................................................uaucacagccugcuugaCgggcu................   1    1    Mm2
......................................................................uUucacagccugcuugaugggcu................   1    1    Mm2
......................................................................uaucacagccugcuugaAgggcu................   3    1    Mm2
......................................................................uaucacagUcugcuugaugggcu................   1    1    Mm2
......................................................................uaucacagccugcuugaugggcA................   2    1    Mm2
......................................................................uaucacagccugcuugaugggcu................   236  0    Mm2
......................................................................uaucacagccugcuugGugggcu................   1    1    Mm2
......................................................................uaucacagccugcuugaugggcG................   7    1    Mm2
......................................................................uaucacagccugcuugaugggcua...............   1    0    Mm2
......................................................................uaucacagccugcuugaugggcuU...............   10   1    Mm2
......................................................................uaucacagccugcuugaugggcuUu..............   2    1    Mm2

..................................uccaaaaaguggcugugaugu......................................................   1    0    Mm1
..................................uccaaaaaguggcugugaugug.....................................................   3    0    Mm1
......................................................................uaucacagccugcuugaugg...................   12   0    Mm1
......................................................................uaucacagccugcuugauggg..................   52   0    Mm1
......................................................................uaucacagccugcuugaugggc.................   69   0    Mm1
......................................................................uaucacagccAgcuugaugggc.................   1    1    Mm1
......................................................................uaucacagccugcuugaugAgc.................   2    1    Mm1
......................................................................uaucacagccugcuugaCgggc.................   1    1    Mm1
......................................................................uaucacagccugcuugaAgggc.................   1    1    Mm1
......................................................................uaucacagccugcuugaugAgcu................   4    1    Mm1
......................................................................uaucacagccugcuugaAgggcu................   1    1    Mm1
......................................................................uaucacagccugcuugaugUgcu................   1    1    Mm1
......................................................................uaucacagccugcuugCugggcu................   1    1    Mm1
......................................................................uaucacagccugcuugaugggcu................   87   0    Mm1
```

```
                                  Star                              Mature
ugguguacuagccgugcauuguauugcuucguauuccaaaaaguggcugugauguguuaaaaucaagucguaucacagccugcuugaugggcuauguggcaauacucuu
......................................................................uaucacagccugcuugaugggcG................ 1 1 Mm1
......................................................................uaucacagccugcuugaGgggcu................ 1 1 Mm1
......................................................................uaucacagccugcuugaugggcA................ 5 1 Mm1

..................................uccaaaaaguggAugugaug....................................................... 1 1 Fm3
..................................uccaaaaaguggcugugaug....................................................... 1 0 Fm3
..................................uccaaaaaguggcugugaugu...................................................... 9 0 Fm3
..................................uccaaaaaguggcugugauguU..................................................... 1 1 Fm3
..................................uccaaaaaguggcugugaugug..................................................... 7 0 Fm3
..................................uccaaaaaguggcugugaugugu.................................................... 4 0 Fm3
..................................uccaaaaaguggcugugauguguu................................................... 2 0 Fm3
......................................................................uaucacagccugcuugau..................... 4 0 Fm3
......................................................................uaucacagccugcuugaug.................... 33 0 Fm3
......................................................................uaucacagccAgcuugaug.................... 3 1 Fm3
......................................................................uaucacagccugcuugaCgg................... 1 1 Fm3
......................................................................uaucacagccugcuugaugg................... 87 0 Fm3
......................................................................uaucacagccugcuugaugU................... 3 1 Fm3
......................................................................uaAcacagccugcuugauggg.................. 1 1 Fm3
......................................................................uaucacagccugcuugaAggg.................. 6 1 Fm3
......................................................................uaucacagcAugcuugauggg.................. 1 1 Fm3
......................................................................Caucacagccugcuugauggg.................. 1 1 Fm3
......................................................................uaucacagccugcuugauggg.................. 644 0 Fm3
......................................................................uaucacagccugcuugauUgg.................. 2 1 Fm3
......................................................................uUucacagccugcuugauggg.................. 1 1 Fm3
......................................................................uaucacagcUugcuugauggg.................. 9 1 Fm3
......................................................................uaucCcagccugcuugauggg.................. 1 1 Fm3
......................................................................uaucacagccugcuugaCggg.................. 5 1 Fm3
......................................................................uaucacagccugcuugCuggg.................. 1 1 Fm3
......................................................................uaucacagccugcuugUuggg.................. 2 1 Fm3
......................................................................uaucacagccGgcuugauggg.................. 1 1 Fm3
......................................................................uGucacagccugcuugauggg.................. 2 1 Fm3
......................................................................uaucacagccugcuugaGggg.................. 6 1 Fm3
......................................................................uaucacagccCgcuugauggg.................. 1 1 Fm3
......................................................................uaucacagccugcuugauCggc................. 1 1 Fm3
......................................................................Naucacagccugcuugaugggc................. 1 1 Fm3
......................................................................uaucacagccugcuugaCgggc................. 4 1 Fm3
......................................................................uaucacagccugcuugUugggc................. 1 1 Fm3
......................................................................uaucacagccugcuugaugggA................. 96 1 Fm3
......................................................................uaucacagccugcuugaugggc................. 614 0 Fm3
......................................................................uaucacagccugcuugaugAgc................. 10 1 Fm3
......................................................................uaucacagccugcuugCugggc................. 1 1 Fm3
......................................................................uauAacagccugcuugaugggc................. 1 1 Fm3
......................................................................uaucacagccAgcuugaugggc................. 3 1 Fm3
......................................................................uaucacagccugcuugGugggc................. 1 1 Fm3
......................................................................uaucacagccugcuugaAgggc................. 1 1 Fm3
......................................................................uaucacagccuAcuugaugggc................. 1 1 Fm3
......................................................................uaucGcagccugcuugaugggc................. 1 1 Fm3
......................................................................uaucacagccugcuugaugUgc................. 1 1 Fm3
......................................................................uaucacagccugcuugauUggc................. 1 1 Fm3
......................................................................uaucacaUccugcuugaugggc................. 1 1 Fm3
......................................................................uaucacagccugcuugaGgggc................. 5 1 Fm3
......................................................................uaucacagcUugcuugaugggc................. 4 1 Fm3
......................................................................uaucacagccugcuugaugggU................. 9 1 Fm3
......................................................................uaucacaAccugcuugaugggc................. 2 1 Fm3
......................................................................uaucacagccugcuugaugggcu................ 675 0 Fm3
......................................................................uaucacagccugcuugaugggAu................ 19 1 Fm3
......................................................................uaucacagccGgcuugaugggcu................ 1 1 Fm3
......................................................................uaucacagccugcuugaCgggcu................ 6 1 Fm3
......................................................................uaucaUagccugcuugaugggcu................ 1 1 Fm3
......................................................................uaucacagccugcuuAaugggcu................ 1 1 Fm3
......................................................................uaucacagccugcuugaugAgcu................ 1 1 Fm3
......................................................................uaucacagccugcuugaugggcA................ 9 1 Fm3
......................................................................uauUacagccugcuugaugggcu................ 1 1 Fm3
......................................................................uaucacagccugcuugCugggcu................ 2 1 Fm3
......................................................................uaucacagcUugcuugaugggcu................ 7 1 Fm3
......................................................................uaucacagccugcuugaGgggcu................ 2 1 Fm3
......................................................................uaucacagccugcuugaAgggcu................ 6 1 Fm3
......................................................................uaucacagccugcuugaugggcua............... 4 0 Fm3
......................................................................uaucacagccugcuugaugggcuU............... 8 1 Fm3
......................................................................uaucacagccugcuugaugggcuUu.............. 2 1 Fm3
```

Star **Mature**

ugguguacuagccgugcauuguauugcuucguauuccaaaaaguggcugugauguguuaaaaucaagucguaucacagccugcuugaugggcuauguggcaauacucuu

..................................uccaaaaaguggcuguga...	1	0	Mm3
..................................uccaaaaaguggcugugaug...	1	0	Mm3
..................................uccaaaaaguggcugugaugu..	25	0	Mm3
..................................uccaaaaaguggcugugaugA..	1	1	Mm3
..................................uccaaaaaguggcugugaugug...	13	0	Mm3
..................................uccaaaaaguggcugugauAug...	1	1	Mm3
..................................uccaaaaaguggcugugauguU...	1	1	Mm3
..................................uccaaaaaguggcuguCaugug...	1	1	Mm3
..................................uccaaaaaguggcugugaugugG..	1	1	Mm3
..................................uccaaaaaguggcugugaugugu..	2	0	Mm3
..................................uccaaaaaguggcuguAaugugu..	1	1	Mm3
....................................caaaaaguggcugugaugu..	1	0	Mm3
....uuaaaaucaagucguaucacagc..............................	1	0	Mm3
....uaucacagccugcuugau.....................	1	0	Mm3
....uaucacagccugcuugaug....................	1	0	Mm3
....uaucacagccugcuugaugg...................	12	0	Mm3
....uaucacagccAgcuugaugg...................	1	1	Mm3
....uaucacagccugcuugaGgg...................	1	1	Mm3
....uaucacagccugcuugaugU...................	1	1	Mm3
....uaucacagccugcuugauggg..................	116	0	Mm3
....uaucacagccugcuugCuggg..................	1	1	Mm3
....uaucacagccugUuugauggg..................	1	1	Mm3
....uaucacagccCgcuugauggg..................	1	1	Mm3
....uaucacagccugcuugauggC..................	1	1	Mm3
....uaucacagccuUcuugauggg..................	1	1	Mm3
....uaucacagccugcuugaAggg..................	1	1	Mm3
....uaucacagUcugcuugauggg..................	1	1	Mm3
....uaucacaUccugcuugaugggc.................	1	1	Mm3
....uaucacGgccugcuugaugggc.................	1	1	Mm3
....uaucacagccugcuugaAgggc.................	1	1	Mm3
....uaucacagccugcuugaugggA.................	10	1	Mm3
....uaucacagccugcuugaCgggc.................	1	1	Mm3
....uaucacagccCgcuugaugggc.................	1	1	Mm3
....uaucacagccugcuugaugAgc.................	2	1	Mm3
....uaucacagccugcuugaugggc.................	147	0	Mm3
....uaucacagccugcuugaugggU.................	2	1	Mm3
....uaucacagccugcuugGugggc.................	1	1	Mm3
....uaucacagccugcuugaugggG.................	1	1	Mm3
....uaucacagUcugcuugaugggc.................	1	1	Mm3
....uaAcacagccugcuugaugggc.................	1	1	Mm3
....uaucacagccugcuugaugggcG................	6	1	Mm3
....uaucacagccugcuugaGgggcu................	1	1	Mm3
....uaucacagccugcuugaugggcu................	117	0	Mm3
....uaucacagccugcuugaugggAu................	1	1	Mm3
....uaucacagcUugcuugaugggcu................	2	1	Mm3
....uaucacagUcugcuugaugggcu................	2	1	Mm3
....uaucacagccugcuugaugggcC................	1	1	Mm3
....uaucacagccugcuugaugggcuU...............	1	1	Mm3
